# Dietary Alaska Pollock Protein Attenuates the Experimental Colitis Induced by Dextran Sulfate Sodium via Regulation of Gut Microbiota and Its Metabolites in Mice

**DOI:** 10.3390/metabo12010044

**Published:** 2022-01-07

**Authors:** Genki Tanaka, Nozomi Hagihara, Ryota Hosomi, Takaki Shimono, Seiji Kanda, Toshimasa Nishiyama, Munehiro Yoshida, Kenji Fukunaga

**Affiliations:** 1 Faculty of Chemistry, Materials, and Bioengineering, Kansai University, 3-3-35, Yamate-cho, Suita 564-8680, Osaka, Japan; k323344@kansai-u.ac.jp (G.T.); k558945@kansai-u.ac.jp (N.H.); hanmyou4@kansai-u.ac.jp (M.Y.); fukunagk@kansai-u.ac.jp (K.F.); 2 Department of Hygiene and Public Health, Kansai Medical University, 2-5-1, Shin-machi, Hirakata 573-1010, Osaka, Japan; shimonot@hirakata.kmu.ac.jp (T.S.); kandas@hirakata.kmu.ac.jp (S.K.); tnishi@takii.kmu.ac.jp (T.N.)

**Keywords:** Alaska pollock protein, colitis, microbiota, short-chain fatty acid, dextran sulfate sodium

## Abstract

Protein derived from fish has not only nutritional properties but also health-promoting properties. Few studies have examined the effect of dietary Alaska pollock protein (APP) on the anticolitis effect reported to be associated with metabolic syndrome (MetS). This study investigated the effect of APP intake on colitis symptoms, gut microbiota, and its metabolites in the experimental colitis mouse model induced by dextran sulfate sodium (DSS). Male C57BL/6J mice were divided into three groups: (1) DSS-untreated mice fed an American Institute of Nutrition (AIN) 93G diet (protein source is casein), (2) DSS-treated mice fed an AIN93G diet, and (3) DSS-treated mice fed an APP diet. After the mice were fed the diets for 21 days, experimental colitis was induced by three cycles of 2% DSS administration for 5 days followed by washouts over the course of 5 days. APP-reduced body weight loss increased the disease activity index, and elevated spleen weight and alleviated colon length shortening and colonic tissue damage. Furthermore, APP altered the structure and composition of the microbiota and short-chain fatty acids in feces. Since APP intake alleviates experimental colitis induced by DSS administration through alterations in the gut microbiota and its metabolites, we deduced that APP would inhibit MetS progression via colitis suppression.

## 1. Introduction

Metabolic syndrome (MetS) refers to obesity combined with dyslipidemia, glucose intolerance, hypertension, and inflammation predisposes to heart disease and stroke [1]. Inflammation is an immune response caused by exposure to pathogens, damaged cells, or toxic compounds [2]. One factor that can cause inflammation is the gut’s environment (i.e., microbiota, intestinal barrier function, and bacteria metabolites) [3]. When the intestinal barrier function is compromised, the intestinal bacteria come into close contact with intestinal epithelium cells, eventually promoting the infiltration of immune cells, the expression of proinflammatory cytokines, and oxidative stress [4]. Increased levels of plasma endotoxins, such as gut microbiota-derived lipopolysaccharides (LPS), have been associated with chronic low-grade inflammation dysfunction and insulin resistance [5]. Dysfunction of the intestinal epithelium is thought to be involved in the early stages of inflammatory bowel disease (IBD) [4]. Epidemiological studies have shown that the incidence of IBD such as ulcerative colitis and Crohn’s disease is increasing [6]. Colitis has been shown to be associated with a number of diseases [7,8]. Among these diseases, MetS is the most common comorbidity, affecting the etiology and treatment of the disease [7]. MetS is also a clinical condition that occurs frequently in older patients with IBD [9]. IBD is considered a lifestyle-related disease, partly because Western dietary habits are thought to reduce the diversity of gut microbiota [10].

The relationship between IBD and some food components is actively being studied [11,12,13]. Meta-analysis has shown a negative correlation between fish intake and the risk of Crohn’s disease, and it has also shown a significant inverse correlation between dietary long-chain *n*-3 polyunsaturated fatty acid (PUFA) and the risk of ulcerative colitis [14]. In experiments involving in vivo subjects with dextran sulfate sodium (DSS)-induced colitis, fish oil containing *n*-3 PUFA has been shown to exert a protective effect against high-fat diets [12]. Considering that *n*-3 PUFA is abundant in fish and has strong physiological and health-promoting benefits, we may have been overlooking other beneficial fish nutrients for the treatment of colitis. In addition to fat, fish is also rich in nutritious protein [15]. Our previous studies have suggested that proteins prepared from several fish species protect against the accumulation of triacylglycerol in the liver [16,17] and reduce the level of serum cholesterol [18]. Other researchers have reported that Alaska pollock protein (APP) exhibits beneficial effects on obesity [19] and muscle hypertrophy [20]. We have recently reported that APP intake suppresses fatty liver disease and enhances insulin sensitivity via changes in the composition of gut microbiota [17,21]. In addition, evidence from mouse models have indicated that fish (*Hypophthalmichthys nobilis*) protein intake can strengthen the integrity of intestinal barriers [22]. Colitis is closely related to the balance of gut microbiota, intestinal barrier function, and bacterial metabolites [23]. Since fish protein intake has been reported to improve intestinal microbiota and barrier function, it is possible that the prevention of colitis by fish intake can be influenced by protein as well as fat (*n*-3 PUFA). Moreover, experimental colitis induced by DSS is linked to metabolic-dysfunction phenotypes including dyslipidemia and hepatosteatosis [24]. Few studies have focused, however, on the effects of APP intake on experimental colitis in mice. Therefore, it was the aim of this study to evaluate the anticolitis effect of the APP diet (APP + DSS group) compared to a casein rich standard rodent diet (American Institute of Nutrition (AIN) 93G, CAS + DSS group) in an experimental DSS induced mouse colitis model.

## 2. Results

### 2.1. Nutritional Compositions of APP

APP is composed mainly of protein, and compared with casein, its composition of amino acids is rich in arginine, alanine, glycine, and aspartic acid (aspartic acid + asparagine) and poor in proline and glutamic acid (glutamic acid + glutamine) (Table 1). The nutritional composition of dried Alaska pollock fillets powder is shown in Table S1. The amount of eicosapentaenoic acid (EPA) and docosahexaenoic acid (DHA) (0.1 g/100 g), which are highly bioactive components, in APP can be reduced by about 80% by defatting with *n*-hexane and ethanol. The resulting modified APP is mainly composed of myosin of about 200 kDa and actin of about 45 kDa (Figure S1).

### 2.2. APP Intake Alleviates Experimental Colitis Symptoms Induced by DSS in Mice

Table S2 shows the mice’s growth parameters and relative organ weights. The water intake of the CAS + DSS group was higher than that of the untreated group. There were no significant differences in food intake and DSS solution intake during the experimental period and DSS administration period, respectively. DSS administration affected the relative liver, small intestine, and cecum weights. On the other hand, APP intake had no effect on the relative organ weights compared with the CAS + DSS group.

**Figure 1:**
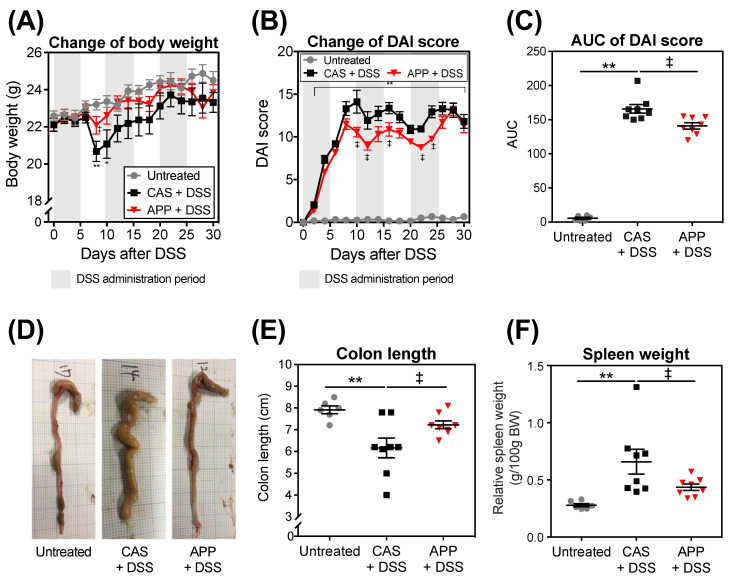
BW, DAI score, colon length, and spleen weight after three repeated cycles of 2% (*w*/*w*) DSS administration via drinking water for five days followed by a washout for five days in mice. (**A**) Change of BW (changes in BW percentage were calculated by dividing the BW on the specified day by the BW at Day 0). (**B**) Change of DAI score. (**C**) AUC of DAI score during DSS administration. (**D**) Representative pictures of colons and (**E**) colon length at Day 30 of DSS administration. (**F**) Relative spleen weight at Day 30 of DSS administration. The values shown are the mean ± SEM (*n* = 6–8 per group). Data were analyzed using the Holm–Sidak multiple comparisons test (**A**, **E**, and **F**) and uncorrected Dunn’s test (**B**,**C**) between the untreated vs. CAS + DSS (* *p* < 0.05 and ** *p* < 0.01) and CAS + DSS vs. APP + DSS (^‡^ *p* < 0.05). CAS, casein; AUC, area under the curve; BW, body weight; DAI, disease activity index; DSS, dodecyl sodium sulfate; SEM, standard error of the mean.

A loss of body weight (BW) and an elevation of the disease activity index (DAI) score were observed in the CAS + DSS and APP + DSS groups from the start of DSS administration (Figure 1A–C). The loss of BW in the APP + DSS group was significantly lower on Day 8 compared with the CAS + DSS group (Figure 1A). The APP + DSS group had a significantly lower DAI score on Day 10, 12, 16, 22, and 24 than the CAS + DSS group (Figure 1B). The area under the curve (AUC) of the DAI score in the APP + DSS group was significantly lower than that of the CAS + DSS group (Figure 1C).

A shortened colon length and an increased spleen weight in DSS-induced mice were used as biological markers of the severity of colitis [25]. The CAS + DSS group revealed a shortened colon length and an increased relative spleen weight compared with the untreated group (Figure 1E,F). The APP intake significantly suppressed shortening of the colon length and an increase in relative spleen weight compared with the CAS + DSS group.

**Figure 2:**
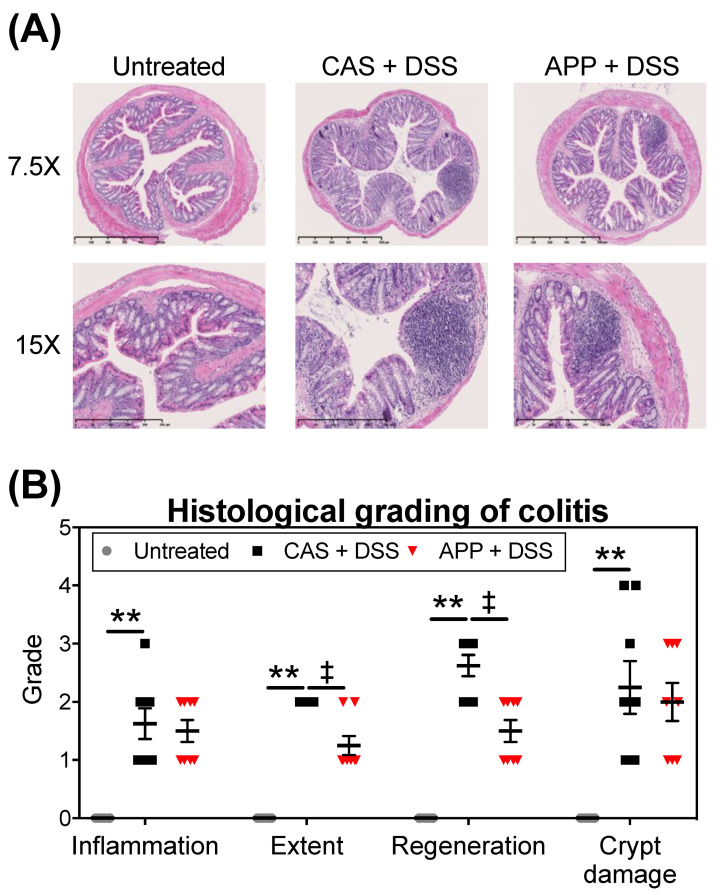
Histological grading of the mice’s colonic tissues after three repeated cycles of 2% (*w*/*w*) DSS administration via drinking water for five days followed by a washout for five days. (**A**) Representative histological sections (scale bar = 500 μm [7.5 X] and 250 µm [15 X]) and (**B**) histological grading in the colon at Day 30 of DSS administration. The colonic tissues were stained with hematoxylin–eosin. The values shown are the mean ± SEM (*n* = 6–8 per group). Data were analyzed using Kruskal–Wallis with uncorrected Dunn’s test between the untreated vs. CAS + DSS (** *p* < 0.01) and CAS + DSS vs. APP + DSS (^‡^ *p* < 0.05).

Three repeated cycles of 2% (*w*/*w*) DSS administration via the mice’s drinking water for five days followed by a washout for five days induced histological colonic damage including inflammation (amount of inflammation), extent (depth of inflammation), regeneration (amount of regeneration), and crypt damage (amount of crypt damage) (Figure 2). The colonic extent and regeneration in the APP + DSS group were significantly attenuated compared to the CAS + DSS group.

**Figure 3:**
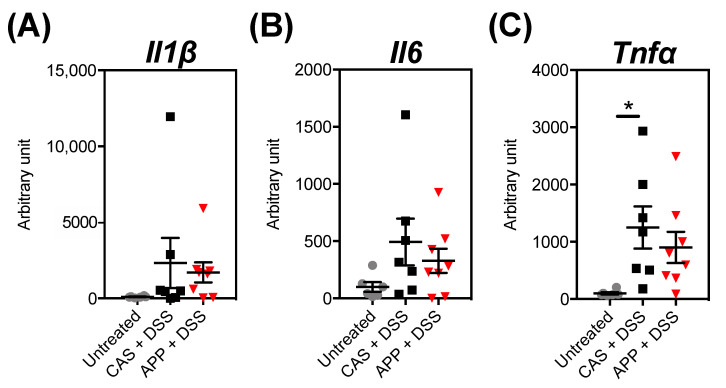
mRNA expression levels in the mice’s colon after three repeated cycles of 2% (*w*/*w*) DSS administration via drinking water for five days followed by a washout for five days. Expression levels of (**A**) *Il1β*, (**B**) *Il6*, and (**C**) *Tnfα* in the mice’s colonic mucosa at Day 30 of DSS administration. The values shown are the mean ± SEM (*n* = 6–8 per group). The levels are expressed relative to those determined using the untreated group (set at 100). Data were analyzed using the Holm–Sidak multiple comparisons test between the untreated vs. CAS + DSS (* *p* < 0.01). *Il1β*, interleukin 1 beta; *Il6*, interleukin 6; *Tnfα*, tumor necrosis factor alpha.

To gain insight on the observed inflammation, the mRNA expression levels of cytokines (interleukin (*Il*)-1β (*Il1β*), *Il6*, and tumor necrosis factor alpha (*Tnfα*)) in the colonic mucosa were measured by quantitative PCR analysis (Figure 3). DSS administration induced the expression level of *Tnfα*. On the other hand, APP intake did not affect the expression levels of *Il1β*, *Il6*, and *Tnfα* compared with the levels observed in the CAS + DSS group.

### 2.3. APP Intake Altered the Composition of the Gut Microbiota of Mice with DSS-Induced Colitis

**Figure 4:**
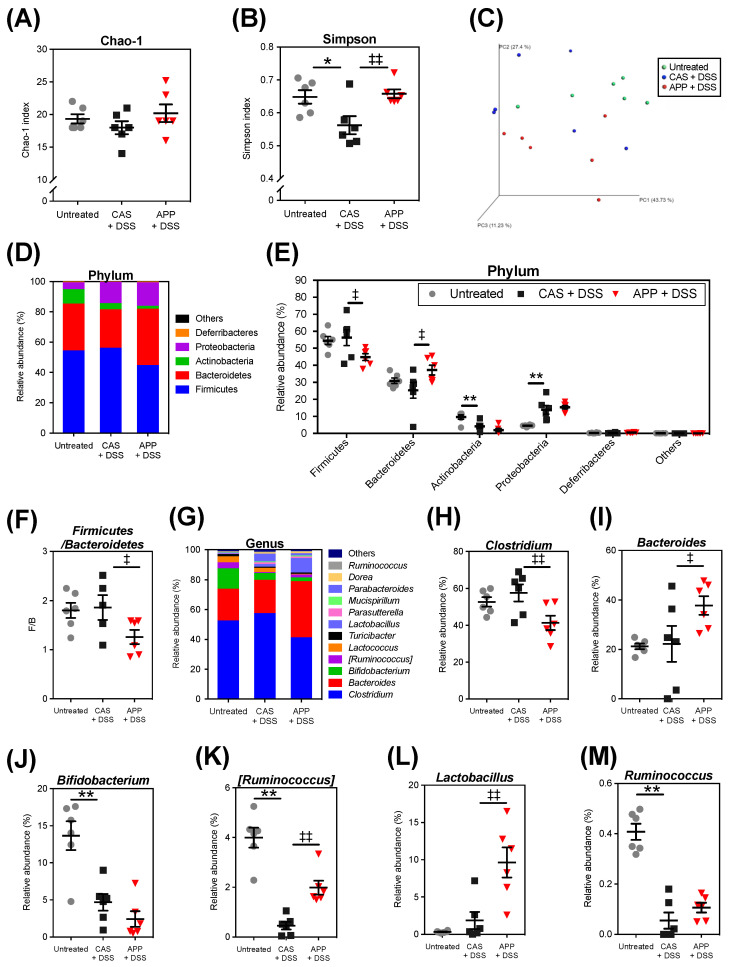
The structure and composition of the microbiota in the mice’s feces after three repeated cycles of 2% (*w*/*w*) DSS administration via drinking water for five days followed by a washout for five days. (**A**) Chao 1 and (**B**) Simpson indices. (**C**) PCoA plot based on the Bray–Curtis index. (**D**) The relative abundance of fecal microbiota at the phylum level. Sorted from the higher relative abundance in the untreated group, and with less than 0.5% of the relative abundance summarized as Others. (**E**) The relative abundance of each bacterium at the phylum level. (**F**) Firmicutes/Bacteroidetes ratio. (**G**) The relative abundance of fecal microbiota at the genus level. Sorted from the higher relative abundance in the untreated group, and with less than 0.5% of the relative abundance summarized as Others. (**H**–**M**) The relative abundance of each bacterium at the genus level. Square brackets ([]) around a genus indicate that the name awaits appropriate action by the researcher for possible transfer to another genus. The values shown are the mean ± SEM (*n* = 6 per group). Data were analyzed using the Holm–Sidak multiple comparisons test between the untreated vs. CAS + DSS (* *p* < 0.05 and ** *p* < 0.01) and CAS + DSS vs. APP + DSS (^‡^ *p* < 0.05 and ^‡‡^ *p* < 0.01). PCoA, principal coordinate analysis.

Because the DSS-induced experimental colitis model alters the structure and composition of the gut microbiota [23], we used next-generation sequencing (NGS)-based 16S rRNA amplicon sequencing to evaluate whether APP intake improves the gut microbiota. There was no significant difference in the number of valid reads among the experimental groups after processing (139,984 ± 22,363, 135,414 ± 21,597, and 137,455 ± 23,267, respectively). The rarefaction curves of α-diversities (Chao-1 and Simpson) of each sample plateaued, indicating that the sequencing depth was sufficient to reflect the diversity of the samples, and the sequencing result was reliable (data not shown). There was no difference in the Chao-1 index among the groups (Figure 4A). The Simpson index of the CAS + DSS group decreased compared with that of the untreated group (Figure 4B). The APP intake attenuated the DSS-induced reduction in the Simpson index. The Bray–Curtis index at the genus level revealed no significant difference in the β-diversity between the groups (Figure 4C).

The relative bacterial abundances at the phylum and genus levels are shown using histograms (Figure 4D,G). DSS administration induced a decrease in the relative abundance of Actinobacteria and an increase in the relative abundance of Proteobacteria (Figure 4E). Compared with the CAS + DSS group, a decrease in the relative abundance of Firmicutes and an increase in the relative abundance of Bacteroidetes were observed in the APP + DSS group. In addition, the Firmicutes/Bacteroidetes (F/B) ratio in the APP + DSS group was significantly lower than that in the CAS + DSS group (Figure 4F). At the genus level, DSS administration induced a decrease in the relative abundances of *Bifidobacterium, [Ruminococcus],* and *Ruminococcus* (Figure 4J,K,M). We observed a lower relative abundance of *Clostridium* and a higher relative abundance of *Bacteroides, [Ruminococcus],* and *Lactobacillus* in the APP + DSS group than in the CAS + DSS group (Figure 4H,I,K,L).

**Figure 5:**
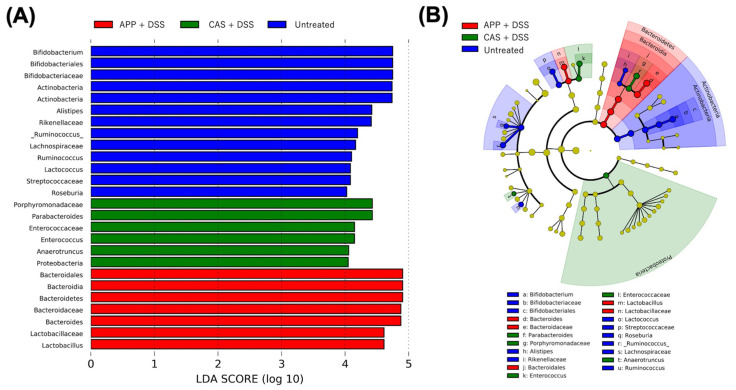
Comparisons of fecal microbiota using the LEfSe analysis. (**A**) Results and (**B**) cladogram using the LEfSe analysis method indicating the phylogenetic distribution of fecal microbiota among the experimental groups. LDA scores (log_10_) > 4 and *p* < 0.05 are listed. LDA, linear discriminant analysis; LEfSe, linear discriminant analysis effect size.

To investigate the effects of DSS administration and APP intake on the key types of fecal microbiota, we used a linear discriminant analysis effect size (LEfSe) analysis to identify the phylotypes with the greatest differences among the groups (Figure 5). The 26 dominant microbiota at each level among the groups were then obtained. At the phylum level, Actinobacteria, Proteobacteria, and Bacteroidetes phylotypes were more abundant in the untreated, CAS + DSS, and APP + DSS groups, respectively. Relative *Enterococcus* abundance in the CAS + DSS group significantly increased. *Enterococcus* species are a common member of intestinal bacteria that are more enriched in the gut of IBD patients [26] and promote colitis in mice IBD models [27].

### 2.4. APP Intake Altered Fecal Short-Chain Fatty Acid (SCFA) Contents/Compositions in Mice with DSS-Induced Colitis

**Figure 6:**
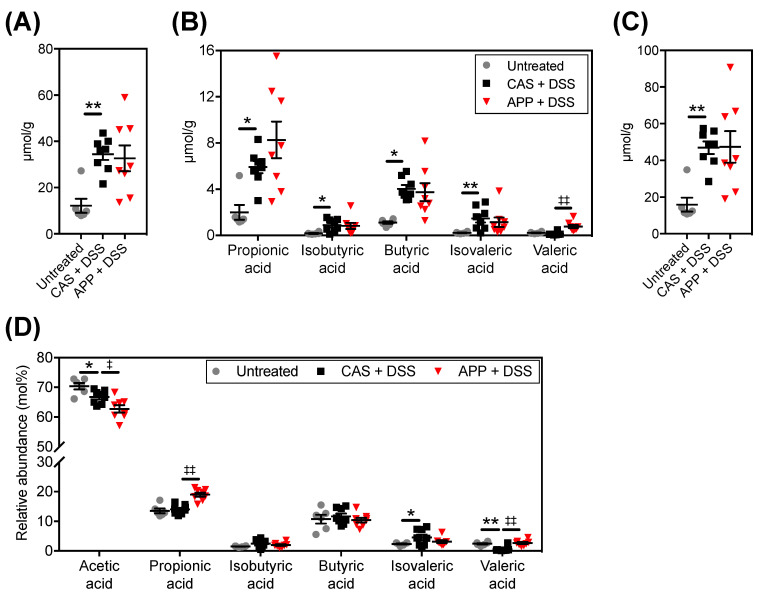
SCFA compositions in the mice’s feces after three repeated cycles of 2% (*w*/*w*) DSS administrated via drinking water for five days followed by a washout for five days. (**A**) acetic acid composition, (**B**) SCFA composition other than acetic acid (**C**) total SCFA content, and (**D**) relative SCFA abundance. The values shown are the mean ± SEM (*n* = 6–8 per group). Data were analyzed using the Holm–Sidak multiple comparisons test between the untreated vs. CAS + DSS (* *p* < 0.05 and ** *p* < 0.01) and CAS + DSS vs. APP + DSS (^‡^ *p* < 0.05 and ^‡‡^ *p* < 0.01). SCFA, short-chain fatty acid.

Bacterial metabolites such as SCFAs in the colon are involved in the pathogenesis of colitis [28]. Therefore, we evaluated the effect of APP intake on the SCFA content and composition in the feces of DSS-induced colitis mice (Figure 6). DSS administration caused an increase in fecal acetic acid, propionic acid, isobutyric acid, butyric acid, isovaleric acid, and total SCFA contents (Figure 6A,B). In addition, the APP + DSS group showed higher concentrations of fecal valeric acid than the CAS + DSS group. Since the cecum weights in the CAS + DSS group were lower than those in the untreated group (Table S3), we hypothesized that this may be due to the enrichment of SCFA in their feces, indicating the relative SCFA composition in feces (Figure 6C). DSS administration reduced the concentration of acetic acid. Moreover, the APP + DSS group showed higher relative propionic acid and valeric acid concentrations and lower relative acetic acid concentrations than the CAS + DSS group.

The correlations between indicators of the severity for colitis and the fecal environment (relative bacteria and SCFA compositions) were analyzed by Spearman’s correlation analysis (Figure S2). The indicators of the severity of colitis significantly correlated with the relative fecal abundance of *[Ruminococcus]*, *Parasutterella*, and *Ruminococcus* and fecal valeric acid composition. Then, the correlations between relative bacteria abundances and fecal SCFA compositions were analyzed by Spearman’s correlation analysis (Figure S3). The relative fecal composition of valeric acid showed a significant positive correlation with the relative fecal abundance of *[Ruminococcus]* (Spearman’s r = 0.5232, *p* = 0.025).

## 3. Discussion

Our previous studies showed that APP has health-promoting benefits, such as improving insulin sensitivity and inhibiting the development of a fatty liver, partly attributable to its effective changes to gut microbiota [17,21]. However, few studies have focused on the effects of APP intake on experimental colitis induced in mice by three repeated cycles of 2% (*w*/*w*) DSS administration via the mice’s drinking water for five days followed by a washout for five days. In this study, we used DSS to induce experimental colitis in order to simulate the clinical colitis [29]. After three repeated cycles of DSS administration, the mice showed watery stools, changes in their BW and DAI scores, shortened colon lengths, increased relative spleen weights, and histological colonic damage (Figure 1 and Figure 2). Other researchers have also reported the induction of the experimental colitis in models using methods similar to those performed in this experiment [29,30]. In the present study, the mice in the CAS + DSS and APP + DSS groups showed phenotypic proof that an experimental colitis model was successfully established. As a result, the indicators of colitis, including decreased BW, shortened colon lengths, increased AUC of DAI scores, relative spleen weight, and histological scores (extent and regeneration), were alleviated in the APP + DSS group compared with the CAS + DSS group, indicating that APP intake suppressed experimental colitis induced by the above-mentioned procedure (Figure 1 and Figure 2).

Although the pathogenesis of colitis is complex, dysbiosis remains one of the most important observations [31]. Both the mouse model of DSS-induced experimental colitis and human ulcerative colitis patients have shown a reduction in the α-diversity of the gut microbiota [32,33]. The Chao-1 index is indicative of the community’s richness, and the Simpson index is indicative of the community’s diversity and evenness [34]. The decreased Simpson index by DSS administration was improved by APP intake (Figure 4B). Thus, APP improved the α-diversity, which was exacerbated by DSS administration, and changed the gut microbiota’s community.

At the phylum level, colitis raises the proportions of the Verrucomicrobia and Proteobacteria phylums, which are thought to be harmful bacteria, in both ulcerative colitis patients and the experimental colitis mouse model [29,35]. In our study, Proteobacteria was enriched in the DSS administration groups, but Verrucomicrobia was not detected (Figure 4E). APP intake decreased the relative abundance of Firmicutes and increased the relative abundance of Bacteroidetes compared with the CAS + DSS group (Figure 4E). The F/B ratio is an indicator of intestinal inflammation, and the F/B ratio increases of DSS-induced colitis in mice [36]. The F/B ratio in the APP + DSS group decreased compared with that of the CAS + DSS group (Figure 4F). In addition, DSS administration did not affect the F/B ratio. Our previous study showed that APP intake decreased the F/B ratio compared to casein intake in normal Wistar rats [21]. Therefore, we deduced that the decrease in the F/B ratio observed in this experiment was not attributable to the improvement of experimental colitis by DSS administration, but rather was attributable to the effect of APP intake. However, it is not clear whether the F/B ratio decreases when APP is given to normal mice, and further studies are needed to clarify this point.

At the genus level, DSS administration reduced the relative abundances of *Bifidobacterium, [Ruminococcus]*, and *Ruminococcus* (Figure 4J,K,M). These reductions are characteristic representations of the gut microbiota in DSS administrated mice from previous reports [37,38]. APP intake decreased the relative abundance of *Clostridium* and increased the relative abundance of *Lactobacillus* (Figure 4H,L). Previous report have indicated that the *Clostridium* genus is involved in increasing the number of inducible regulatory T cells in the colon [39]. In addition, previous study has shown that relative abundance of *Clostridium* is decreased in patients with IBD [40]. Our previous study showed that APP intake reduced the relative abundance of *Clostridium* compared with casein intake in normal Wistar rats [21]. The decrease in the relative abundance of *Clostridium* observed in the APP + DSS group is thought to be due to the effects of APP intake, and the extent to which this decrease in the relative abundance of *Clostridium* affects the pathogenesis of colitis is not known at this time. *Lactobacillus*, a typical probiotic bacterium, is widely recognized for its immunomodulating and health-promoting properties that help prevent and treat a variety of allergic, infectious, and inflammatory diseases [41]. In addition, oral administration of *Lactobacillus* species could increase intestinal barrier function and tight junction (TJ) protein expression in mice and piglets [42,43]. Previous studies have shown that high-fat diets with supplemental fish (*Hypophthalmichthys nobilis*) protein increased TJ proteins (zonula occludens-1, occludin, and claudin-1) expression levels in the colon and the relative abundance of *Lactobacillus* compared with high-fat diets with casein [22]. Some reports have shown that TJ protein levels play an important role in the epithelium barrier and prevent the invasion of pathogenic microorganisms. Furthermore, studies have indicated that the abnormal expression of TJ protein correlates with IBD [44]. To clarify whether colonic TJ protein is involved in the suppression of colitis by APP intake, it was necessary to examine the effect of APP intake on colonic TJ protein expression.

SCFAs, metabolites of gut microbiota, are released when bacteria utilize indigestible carbohydrates and proteins, and they serve as the primary energy substrate for colonic epithelial cells [45]. SCFAs are closely associated with intestinal anti-inflammation [46] and anticancer effects [47], and they have a potential impact on colon disease [48]. Although DSS-induced experimental colitis mice showed a reduction in the content of SCFAs in their colons and feces [49], the total fecal content of SCFAs increased in mice administered DSS (Figure 6B). It has been reported that patients with inflamed colonic mucosa [50] and active ulcerative colitis [51] have impaired absorption and metabolism of SCFAs. Therefore, the increased fecal total SCFA content observed in mice administered DSS may be related to colonic dysfunction and the progression of colitis. Bacteroidetes is one of the most abundant phyla in the gut and produces mainly acetate and propionate [52]. The reason why fecal total SCFA content of the APP + DSS group did not decrease despite the suppression of experimental colitis by APP may be due to the increased relative abundance of Bacteroidetes producing acetic acid and propionic acid, which account for most of the SCFAs.

The relative fecal composition of valeric acid negatively correlated with the indicators of the severity of colitis (Figure S2). Valeric acid has been identified as a potential therapeutic target [53]. Unlike other main SCFAs such as acetic acid, propionic acid, and butyric acid, the role of valeric acid in gut health is not fully understood. Limited studies have found that valeric acid has a beneficial effect on the development of colitis through the inhibition of histone deacetylase [54] and promotes the growth of intestinal epithelium cells [55]. In addition, intestinal microflora are thought to produce valeric acid [56]. The relative fecal composition of valeric acid positively correlated with the relative fecal abundance of *[Ruminococcus]* (Figure S3). Other reports also have indicated that valeric acid levels positively correlated with the relative abundance of *[Ruminococcus]* in human studies [11,57]. To the best of our knowledge, there have been no reports on *[Ruminococcus]* producing valeric acid.

The indicators of the severity of colitis significantly correlated with the relative fecal abundance of *[Ruminococcus]*, *Parasutterella*, and *Ruminococcus* (Figure S2). APP intake reversed the reduction in the relative abundance of *[Ruminococcus]* in DSS-induced colitis mice (Figure 4J). Therefore, it is possible that *[Ruminococcus]* is involved in the suppression of colitis by APP intake. In general, dysbiosis in IBD patients and in the experimental colitis model mouse are associated with a decrease in *Ruminococcus* bacteria, which are SCFA-producing bacteria [13,58]. *Ruminococcus* has been linked to the intake of high fiber and resistant starch, which contribute significantly to butyrate production in the colon [59], but fecal butyric acid content/composition was not altered by APP intake (Figure 6A,C). This study did not reveal how *[Ruminococcus]* is associated with DSS-induced colitis, but it is possible that *[Ruminococcus]* is a key bacterial player in the improvement of colitis by APP intake.

Our results propose that APP intake suppresses experimental colitis induced by three repeated cycles of 2% (*w*/*w*) DSS administration via drinking water for five days followed by a washout for five days. This could be linked to the amino acid composition of APP. Arginine has been shown to protect cells from apoptosis caused by LPS-induced oxidative damage in cell studies [60]. Aspartic acid supplies energy to intestinal epithelial cells, helps to maintain the integrity of the intestinal mucosa through mitochondrial oxidation, regulates the AMP-activated protein kinase–mammalian target of the rapamycin pathway, and promotes intestinal epithelial cell proliferation [61]. Arginine and aspartic acid are more abundant in APP than in casein (Table 1). However, dietary proteins are hydrolyzed by digestive enzymes in the gastrointestinal tract; therefore, it is impetuous to consider the amino acid composition of intact proteins. To clarify the components of APP that inhibit experimental colitis, it is necessary to first evaluate what components reach the colon using an in vitro digestion model.

There is a limitation in the present study. Previous studies reported that the SCFA composition differs between cecum content and feces because SCFA is used as an energy substrate by colonic epithelial cells [62]. This study evaluated SCFA in feces, not in cecal contents, so the changes in fecal SCFA composition due to APP intake do not apply to the cecal SCFA composition. However, since the human study evaluates fecal SCFA composition, we believe that these data are useful for comparison with the human study.

## 4. Materials and Methods

### 4.1. Materials and Experimental Diets

Alaska pollock fillets obtained from a local supermarket were chopped into small pieces, freeze-dried (FDU-1200, EYELA, Tokyo, Japan), and ground using a Waring blender (GM200; Retsch Technology GmbH, Haan, Germany), residues were named dried Alaska pollock fillets powder. Dried Alaska pollock fillets powder washed at least six times with *n*-hexane/ethanol (1:1) to remove fats and residues were air-dried and then stored at −35 °C. The resulting products were named APP. Other ingredients of the diet were purchased from Oriental Yeast Co., Ltd. (Tokyo, Japan) and Fujifilm Wako Pure Chemical Co. (Osaka, Japan). DSS was purchased from MP Biomedicals (Irvine, CA, USA). Table S3 shows the ingredients of the experimental diets based on the AIN93G formula [63]. All special level reagents were obtained from Nacalai Tesque, Inc. (Kyoto, Japan) and Merck KGaA (Darmstadt, Germany).

### 4.2. Nutritional Compositions of the Experimental Proteins (Casein and APP)

Moisture, ash, crude fat, and crude protein of casein, APP, and dried Alaska pollock fillets powder were determined in accordance with a previous study [64]. Amino acid compositions were determined by Japan Food Research Laboratories (Tokyo, Japan). EPA and DHA contents were determined using a gas chromatograph (GC) with flame ionization detection (GC-FID) (GC-2014; Shimadzu Co., Kyoto, Japan) equipped with an Omegawax^®^ capillary column (Merck KGaA), as described in our previous study [65]. The protein molecular weight profile was conducted by sodium dodecyl sulfate-polyacrylamide gel electrophoresis (SDS-PAGE) in accordance with a previous study [17].

### 4.3. Animal Experiments

Male four-week-old C57BL/6J specific pathogen-free (SPF) mice were obtained from Japan SLC, Inc. (Hamamatsu, Japan). The mice were housed alone in plastic cages under SPF conditions and a stable air-conditioned room (temperature: 21–23°C; illuminated, 08:00–20:00) and had free access to food and water. After acclimatization for seven days on the CAS diet, the mice were divided to three groups (untreated, CAS + DSS, and APP + DSS; six mice/DSS-untreated group and eight mice/DSS-treated groups) with similar mean BW and standard deviation. The untreated and CAS + DSS groups received the CAS diet and the APP + DSS group received the APP diet. BW and food intake were measured every two days for three weeks. After three weeks of rearing, the mice were exposed to three repeated cycles of 2% (*w*/*w*) DSS solution for five days followed by a washout for five days (with distilled water). The DAI score [66], which is a composite score derived from a compilation of relative BW loss, stool consistency, and occult blood in the stool, was assessed every two days at 10:00 for 30 days from the start of DSS administration. Figure S4 shows the time schedule of this animal experiment. The humane endpoint was set as more than 20% BW loss compared with the BW at the start of DSS administration, and there were no applicable mice.

At Day 30 of DSS administration, feces samples were collected from each mouse cage. The mice (not fasting) were then euthanized under isoflurane anesthesia (9:00–11:00). The livers, kidneys, spleens, small intestines, cecums, and colons were quickly removed from the mice and weighed, and the colons were also measured for length. The distal colons, which had their colonic contents washed out with cold saline, were fixed in a 10% formalin solution.

### 4.4. Histopathological Analysis

Distal colon sections fixed in 10% formalin were embedded in paraffin and 5-µm sections were stained with hematoxylin–eosin. Histological analyses for inflammation, extent, regeneration, and crypt damage were performed by a pathologist in accordance with a previous study [67].

### 4.5. mRNA Expression Analysis

RNA isolation and cDNA synthesis of the colonic mucosa were conducted using the TRIzol^®^ reagent (Thermo Fisher Scientific, Inc., Waltham, MA, USA) and GoScript^TM^ Reverse Transcription System (Promega Co., Madison, WI, USA), respectively. Each gene mRNA expression was measured in duplicate using a Thermal Cycler Dice^®^ Real Time System (Takara Bio, Inc., Kusatsu, Japan) and GoTaq^®^ qPCR Master Mix (Promega Co.). The PCR primer sequences were as follows: Fwd: 5′- AAATGGTGAAGGTCGGTGTG -3′ and Rev: 5′- AATCTCCACTTTGCCACTGC -3′ for glyceraldehyde-3-phosphate dehydrogenase (*Gapdh*); Fwd:5′- AACAAACCCTGCAGTGGTTC -3′ and Rev: 5′- ACTTGCACAAGGAAGCTTGG -3′ for *Il1β*; Fwd:5′- CCAGAAACCGCTATGAAGTTCC -3′ and Rev: 5′- ACCAGCATCAGTCCCAAGAAG-3′ for *Il6*; Fwd:5′- TGAGGTCAATCTGCCCAAGTAC -3′ and Rev: 5′- TCCCTTCACAGAGCAATGACTC-3′ for *Tnfα*. Results were quantified using a comparative method and were expressed as a relative level after normalization to the level of *Gapdh* expression.

### 4.6. Fecal Short-Chain Fatty Acid (SCFA) Compositions

The SCFA composition in the feces at Day 30 of DSS administration was analyzed using a GC-FID (GC-2014, Shimadzu Co., Kyoto, Japan) in accordance with our previous study [68].

### 4.7. The 16S rRNA Amplicon Sequence and Bioinformatics

Each of the six samples in the experimental groups were randomly selected for this experiment. Total fecal DNA was extracted using an ISOSPIN Fecal DNA (Nippon Gene Co., Ltd., Tokyo, Japan) in accordance with the manufacturer’s instructions. To identify bacteria from the extracted fecal DNA, V2-4-8 and V3-6, 7–9 hypervariable regions of the 16S rRNA gene were amplified by PCR using the Ion 16S^TM^ Metagenomics Kit (Thermo Fisher Scientific, Inc.). The 16S rRNA amplicon sequence analysis was performed using a NGS system (Ion PGM^TM^, Thermo Fisher Scientific, Inc.) as described in our previous report [69]. The raw sequence data were analyzed with Ion Reporter Software using workflow of Metagenomics 16S w1.1 ver. 5.14 (Thermo Fisher Scientific, Inc.). The α-diversity indices (Chao-1 and Simpson) at the genus level for each sample were obtained from the Ion Reporter Software. The β-diversity at the genus level was estimated and visualized with principal coordinate analysis (PCoA) based on the Bray–Curtis index using Ion Reporter Software. LEfSe was performed to identify high-dimensional biomarkers [70] and to characterize the differences among the experimental groups at the genus or higher taxonomic levels using Galaxy (http://huttenhower.sph.harvard.edu/galaxy/ (accessed on 12 November 2021)).

### 4.8. Statistical Analyses

Data were showed as the mean values and standard errors of the mean (SEM). Statistical analyses between the untreated and CAS + DSS groups and between the CAS + DSS and APP + DSS groups involved the Kruskal–Wallis test followed by the uncorrected Dunn’s test for DAI and histopathological scores and the one-way analysis of variance followed by the Holm–Sidak multiple comparisons test for the other data sets. A *p*-value of less than 0.05 was considered statistically significant and a 0.05 ≤ *p*-value < 0.10 was considered to have statistical tendency. The analyses were performed using GraphPad Prism software version 7.0 d (GraphPad Software, Sandiego, CA, USA).

## 5. Conclusions

APP has protective effects against experimental colitis induced by three repeated cycles of 2% (*w*/*w*) DSS administration via drinking water for five days followed by a washout for five days by several means, including by improving the composition of gut microbiota and its metabolites, such as SCFAs. Through systematic data analysis and literature review, we have identified the *[Ruminococcus]* genera and valeric acid as key factors associated with the alleviation of symptoms of experimental colitis by APP intake. These results will contribute to the understanding of APP’s function in alleviating experimental colitis and its inhibition of MetS progression via colitis suppression. Therefore, APP has the potential to be used as a functional food material for the prevention of colitis and MetS.

## Figures and Tables

**Table 1:** Nutritional composition of dietary proteins (casein and APP).

Nutritional Composition			Dietary Proteins	
			Casein ^1^	APP ^2^
Water (g/100 g)			2.6	1.0
Ash (g/100 g)			1.8	6.3
Crude protein (g/100 g)			93.7	92.0
	Amino acid composition (wt% of total amino acid)			
		Arginine	3.56	6.66
		Lysine	8.04	10.16
		Histidine	2.68	2.04
		Phenylalanine	4.88	4.17
		Tyrosine	5.32	3.72
		Leucine	9.03	8.67
		Isoleucine	5.23	5.03
		Methionine	2.53	3.29
		Valine	6.23	5.35
		Alanine	3.06	6.41
		Glycine	1.81	4.65
		Proline	10.6	3.36
		Glutamic acid ^3^	21.08	16.20
		Serine	4.92	4.16
		Threonine	3.85	4.36
		Aspartic acid ^4^	6.81	10.65
		Cysteine	0.37	1.12
Crude fat (g/100 g)			1.3	0.7
		EPA + DHA (g/100 g)	ND	0.1

^1^ Casein is contained as main protein in AIN93G (untreated and CAS + DSS group’s diet). ^2^ Alaska pollock fillets were chopped into small pieces, freeze-dried, and washed with *n*-hexane/ethanol (1:1) to remove most of fat. Residues were air-dried, ground using a Waring blender, and were named APP. APP is contained as main protein in APP + DSS group’s diet. ^3^ Glutamic acid + glutamine. ^4^ Aspartic acid + asparagine. APP, Alaska pollock protein; EPA, eicosapentaenoic acid; DHA, docosahexaenoic acid; ND, not detected.

## Data Availability

The data presented in this study are available on request from the corresponding author. The data are not publicly available due to have not setup a public archive platform for data sharing.

## Supplementary Materials

The following are available online at https://www.mdpi.com/article/10.3390/metabo12010044/s1, Figure S1: sodium dodecyl sulfate-polyacrylamide gel electrophoresis (SDS-PAGE) patterns of casein and APP, Figure S2: heatmap representation of Spearman’s correlation coefficient between the indicators of the severity for colitis and the fecal environment (relative bacteria and SCFA compositions), Figure S3: heatmap representation of Spearman’s correlation coefficient between relative bacteria abundances and fecal SCFA compositions, Figure S4: schematic diagram of DSS-induced colitis, Table S1: nutritional composition of dried Alaska pollock fillets powder, Table S2: composition of the experimental diets, Table S3: growth parameters and organs weights.
