# Supplementary material for: Dietary Alaska Pollock Protein Attenuates the Experimental Colitis Induced by Dextran Sulfate Sodium via Regulation of Gut Microbiota and Its Metabolites in Mice
Source: Metabolites. 2022 Jan 7;12(1):44. doi: 10.3390/metabo12010044 (PMC8779829; doi:10.3390/metabo12010044)
Supplement: Supplementary file 1 [file metabolites-12-00044-s001.zip › metabolites-1526596-supplementary.pdf]

Supplemental Information

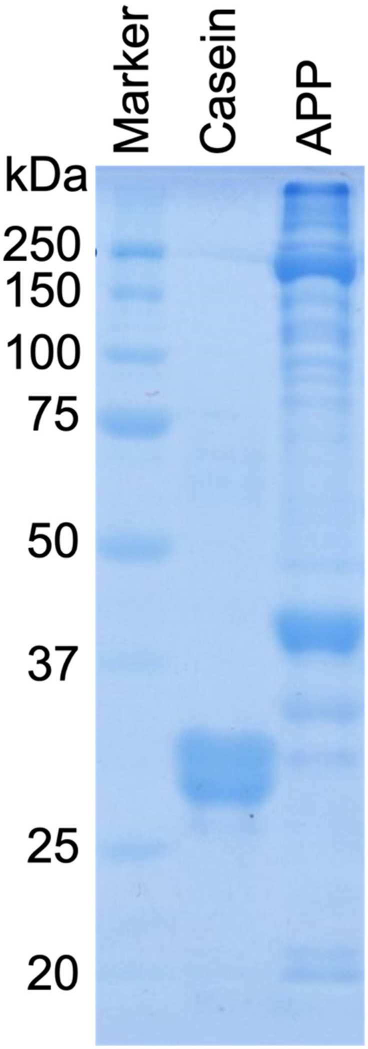

Figure S1. Sodium dodecyl sulfate-polyacrylamide gel electrophoresis (SDS-PAGE) patterns of casein and APP. SDS-PAGE was performed using 12.5% polyacrylamide separation gel; lane (marker), molecular weight marker; lane (casein), casein; lane (APP), Alaska pollock protein.

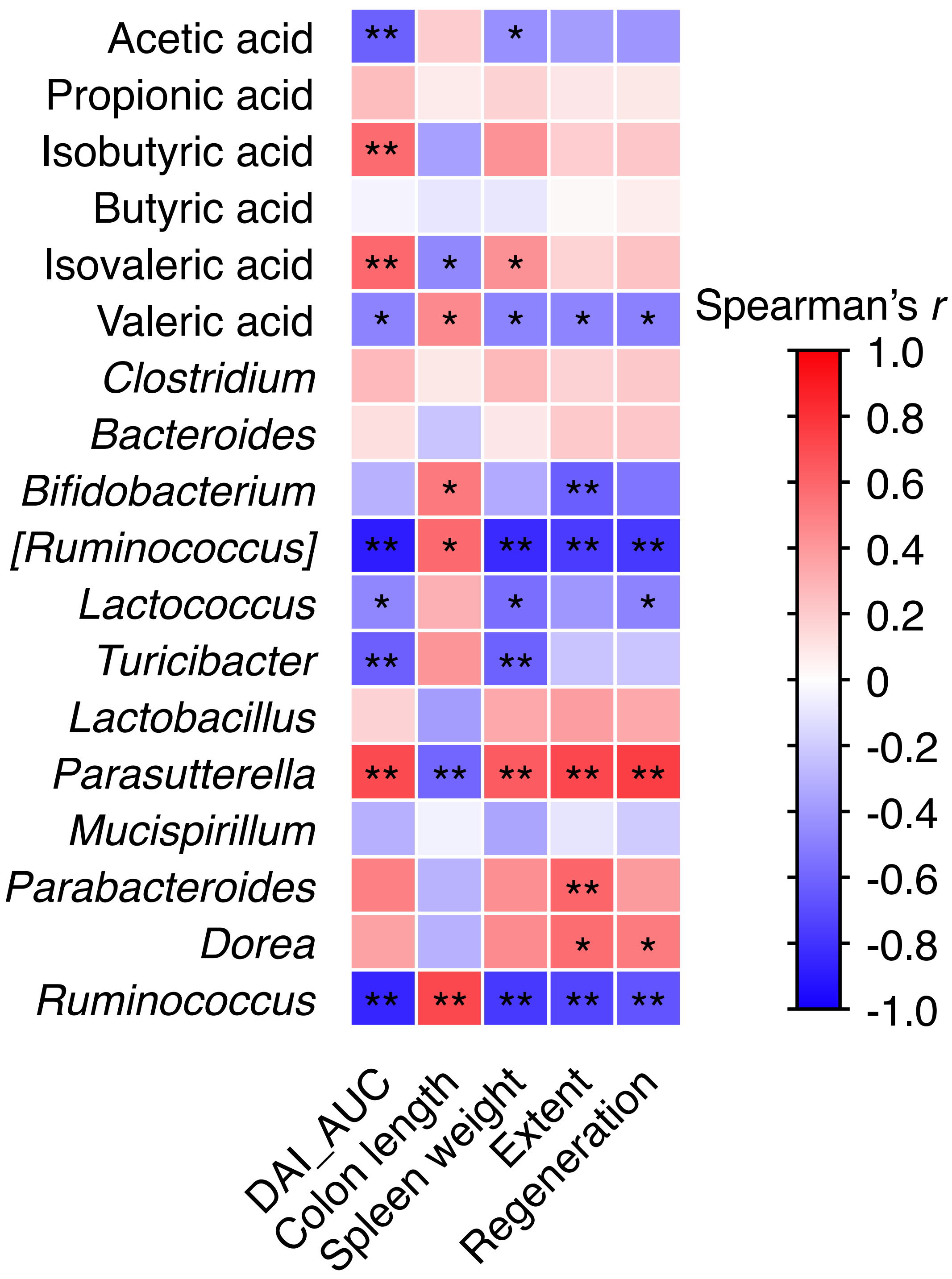

Figure S2. Heatmap representation of Spearman's correlation coefficient between the indicators of the severity for colitis and the fecal environment (relative bacteria and SCFA compositions). Red: position correlations; blue: negative correlations. *$p < 0.05$ and **$p < 0.01$. SCFA, short-chain fatty acid; DAI, disease activity index; AUC, area under the curve.

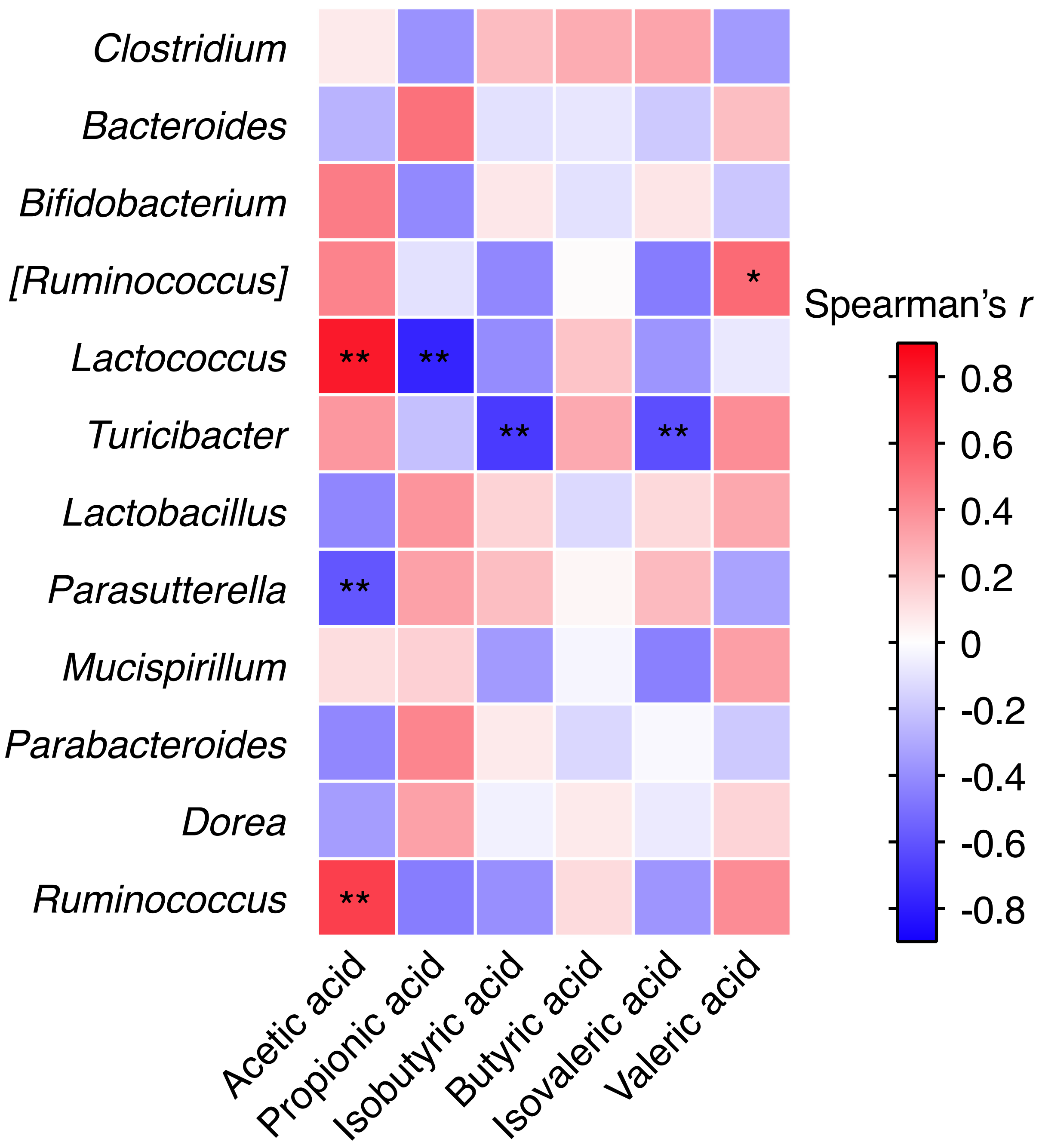

Figure S3. Heatmap representation of Spearman's correlation coefficient between relative bacteria abundances and fecal SCFA compositions. Red: position correlations; blue: negative correlations. *$p < 0.05$ and **$p < 0.01$.

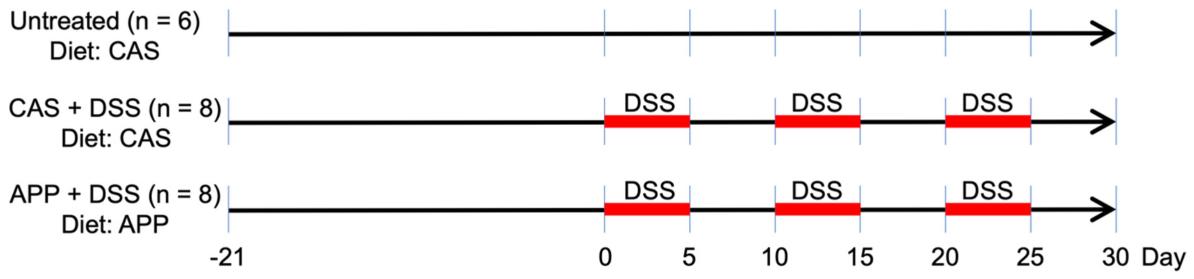

Figure S4. Schematic diagram of DSS-induced colitis.
APP, Alaska pollock protein; CAS; casein; DSS, dodecyl sodium sulfate.

Table S1. Nutritional composition of dried Alaska pollock fillets powder.

Nutritional composition	Dried Alaska pollock fillets powder
Water (g/100g)	1.4
Ash (g/100g)	5.2
Crude protein (g/100g)	87.2
Crude fat (g/100g)	3.6

Table S2 Growth parameters and organs weights.

	Experimental groups		
	**Untreated**	**CAS + DSS**	**APP + DSS**
Growth parameters			
Food intake (g/day)	2.97 ± 0.04	2.95 ± 0.09	2.96 ± 0.04
Water intake (g/day)	5.85 ± 0.16	6.62 ± 0.13 **	6.56 ± 0.10
DSS solution intake (g/day)	-	7.43 ± 0.14	7.35 ± 0.13
Organ weights (g/100g BW)			
Liver	3.99 ± 0.12	4.96 ± 0.35 **	5.12 ± 0.21
Kidney	1.23 ± 0.08	1.23 ± 0.07	1.17 ± 0.02
Small intestine	4.02 ± 0.13	4.97 ± 0.38 **	4.34 ± 0.20
Cecum	1.40 ± 0.10	0.86 ± 0.05 **	0.72 ± 0.09

The values shown are the mean ± SEM (n = 6–8 per group). Data were analyzed using the Holm–Sidak multiple comparisons test between the untreated vs CAS+DSS (**$p < 0.01$). BW, body weight; SEM, standard error of the mean.

Table S3. Composition of the experimental diets.

Ingredients	Experimental groups	
	CAS	APP
	g/kg	
Casein	200	
APP		203.7
Dextrinized corn starch	132	132
Corn starch	398.686	393.786
Sucrose	100	100
Cellulose	50	50
L-Cystine	3	3
Choline bitartrate	2.5	2.5
AIN-93G mineral mixture	35	35
AIN-93 vitamin mixture	10	10
Soybean oil	68.8	70
tert-Butylhydroquinone	0.014	0.014

AIN, American Institute of Nutrition; APP, Alaska pollock protein; CAS, casein.
